# The Effect of Novel Heterocyclic Compounds on Cryptococcal Biofilm

**DOI:** 10.3390/jof3030042

**Published:** 2017-07-20

**Authors:** Maya Korem, Sarah Kagan, Itzhack Polacheck

**Affiliations:** Department of Clinical Microbiology and Infectious Diseases, Hadassah-Hebrew University Medical Center, Jerusalem 91120, Israel; mayak@hadassah.org.il (M.K.); sarah.kagan@mail.huji.ac.il (S.K.)

**Keywords:** *Cryptococcus*, biofilm, quorum sensing, thiazolidinedione derivative, succinimide derivative, auto-inducer

## Abstract

Biofilm formation by microorganisms depends on their communication by quorum sensing, which is mediated by small diffusible signaling molecules that accumulate in the extracellular environment. During human infection, the pathogenic yeast *Cryptococcus neoformans* can form biofilm on medical devices, which protects the organism and increases its resistance to antifungal agents. The aim of this study was to test two novel heterocyclic compounds, S-8 (thiazolidinedione derivative, TZD) and NA-8 (succinimide derivative, SI), for their anti-biofilm activity against strains of *Cryptococcus neoformans* and *Cryptococcus gattii*. Biofilms were formed in a defined medium in 96-well polystyrene plates and 8-well micro-slides. The effect of sub-inhibitory concentrations of S-8 and NA-8 on biofilm formation was measured after 48 h by a metabolic reduction assay and by confocal laser microscopy analysis using fluorescent staining. The formation and development of cryptococcal biofilms was inhibited significantly by these compounds in concentrations below the minimum inhibitory concentration (MIC) values. These compounds may have a potential role in preventing fungal biofilm development on indwelling medical devices or even as a therapeutic measure after the establishment of biofilm.

## 1. Introduction

*Cryptococcus neoformans* is the etiologic agent of meningo-encephalitis, a life-threatening mycosis mainly affecting immunocompromised patients [1], especially acquired immune deficiency syndrome (AIDS) sufferers. The disease has a worldwide distribution and is caused by two pathogenic members of the genus *Cryptococcus*, *C. neoformans* and *C. gattii*. Estimates of the annual global burden of disease and the case fatality rate are nearly 1 million cases and around 625,000 deaths, respectively [2,3]. Cryptococcal infections are typically characterized by the occurrence of large tissue concentrations of organisms with accompanying large amounts of capsular polysaccharides, mainly glucuronoxylomannan (GXM), a known virulence factor in *C. neoformans* [4]. *C. neoformans* also has the ability to adhere and form biofilms on polystyrene plates [5] and medical devices [6,7,8,9] such as polytetrafluoroethylene prosthetic dialysis fistulas [8], prosthetic cardiac valves [7], and ventriculoatrial shunt catheters that may be used to treat *Cryptococcus* central nervous system (CNS) infection [6]. These biofilms consist of a complex network of yeast cells enmeshed in a substantial amount of polysaccharide matrix [5,10], and are formed following a discrete sequence of events, including fungal surface adhesion, micro-colony formation, and matrix production [5]. Studies of acapsular mutants have demonstrated no biofilm formation, while restoration of the capsule resulted in biofilm production, implying the critical role of capsular polysaccharides in this process [5]. Cryptococcal biofilms have been described as a protective niche against microbial predators in nature and, like other microbe-forming biofilms, *C. neoformans* biofilms are resistant to antimicrobial agents and host defense mechanisms, causing significant morbidity and mortality [11].

Increased resistance of the biofilm to antimicrobial therapy in contrast to the planktonic forms [12,13] is multifactorial and involves limited drug penetration due to the high density of extracellular matrix, decreased growth rate due to nutrient limitation, activation of the general stress response, and the existence of subpopulation of cells within the biofilm, known as persisters, that are preserved by antimicrobial pressure [14,15]. Fungal biofilm formation, integrity, and resistance to antimicrobial agents is dependent on quorum sensing (QS) [16], which is mediated by exogenous signaling molecules, called autoinducers (AIs), that accumulate during cell growth in the extracellular environment and, after reaching threshold concentrations, induce changes in microbial gene expression that trigger population cooperation [17,18,19,20,21,22]. The presence of QS in *C. neoformans* was demonstrated by adding medium in which *C. neoformans* had previously grown to fresh cultures. This resulted in the faster growth of *C. neoformans* both as planktonic cells and biofilms. In addition, there was increased production of two virulence factors that the organism uses to thrive in the host: capsular carbohydrates and melanin pigment [4].

Thiazolidinedione (TZD) and succinimide (SI) are heterocyclic compounds proposed as potential QS inhibitors in *Vibrio harveyi*, probably affecting autoinducer I-2 (AI-2), a mixture of signaling molecules thought to function as a universal signal for interspecies communication [23] Blocking of AI-2 QS in *V. harveyi* was shown to be mediated by decreasing the DNA binding ability of LuxR, a key enzyme in the production of AI-2 [24].

Two novel compounds, TZD derivative S-8 and SI derivative NA-8, were found to be effective anti-biofilm agents when tested on *Candida albicans* [25]. This anti-*Candida* biofilm effect was found to be multifactorial, affecting morphogenesis through the yeast to hyphal form transition and the induction of true hypha, cell wall composition, substrate attachment, sterol distribution during germination, and biofilm viability [26,27,28]. S-8 was also recently revealed to be an inhibitor of *C. neoformans* growth through the inhibition of Cdc25 phosphatase and cell cycle arrest [29]. The aim of this study was to investigate the effect of these compounds on biofilm formation by *C. neoformans* and *C. gattii* in an in vitro model of biofilm.

## 2. Materials and Methods

### 2.1. Cryptococcus Strains and Growth Conditions

*Cryptococcus neoformans* strain H-99 (serotype A) and *Cryptococcus gattii* strain R-272 (serotype B) were obtained from CBS-KNAW Collections (Utrecht, The Netherlands). The strains were sub-cultured from sterile vials onto Sabouraud dextrose agar (Novamed, Jerusalem, Israel). The incubation temperature throughout was 35 °C. *Cryptococcus* cells taken from the plate were grown in Sabouraud dextrose broth for 24 h at 30 °C in a rotary shaker at 150 rpm to reach early stationary phase. Minimal growth medium containing 10 g/L glucose, 1 g/L asparagine, 3 g/L KH_2_PO_4_, 1 g/L MgSO_4_·7H_2_O, and 25 mg/L thiamine, pH 6.5, was used for the purpose of biofilm formation [5].

### 2.2. Determination of Antifungal Susceptibility

Minimum inhibitory concentration (MIC) values of the tested compounds (S-8 and NA-8) were determined according to the Clinical Laboratory Standard Institute (CLSI) recommendation for the microbroth dilution method of antifungal susceptibility testing of yeast (M-27-A3). The MIC values were also determined in the minimal growth medium.

### 2.3. Biofilm Formation

Fungal cells were collected by centrifugation, washed twice with phosphate buffer saline (PBS), counted using a hemacytometer, and suspended at 10^7^ cells/mL in a minimal growth medium [5]. For the purpose of measuring metabolic activity, 100 µL of each suspended strain was added into individual wells of polystyrene 96-well plates (Nunc, Roskilde, Denmark) with 100 µL of the tested compounds at 0 (control), 1/4, and 1/16 of the determined MIC in the same media. The tested compounds, TZD and SI, were synthesized according to a previously described method [25] and prepared in dimethyl sulfoxide (DMSO) as stock solutions at a concentration of 20 g/L. The final concentration of the tested compounds in the tested media was 1 g/L. DMSO 0.5% was used as a no drug control. The same method was performed for the experiments with confocal microscopy, except that for these experiments we used 8-well ibiTreat μ-Slides (Ibidi, Munich, Germany) with 200 µL of each suspended strain and 200 µL of the test compounds at concentrations of 0, 1/4, and 1/16 of the MIC. The plates were incubated at 35 °C ambient CO_2_ without shaking. After 24 h, media were replaced with fresh media containing the test compounds, and incubated for an additional 24 h. The cells were then washed with the same media to remove non-adhered cryptococcal cells. True biofilm was represented by cells that remained attached to the plastic surface after replacing the media. The effect of S-8 and NA-8 on biofilm formation was assessed after 48 h by a metabolic reduction assay (XTT cell proliferation kit), confocal microscopic examination (CM) of the biofilms using fluorescent staining (live/dead stain), and analysis of biofilm thickness by Confocal Laser Scanning Microscopy (CLSM). Each compound was tested in triplicate on two separate experiments.

### 2.4. Measuring of Biofilm Metabolic Activity by XTT Reduction Assay

We used the XTT-based cell proliferation kit (Biological Industries, Beit Haemek, Israel). The plates were read at 450 nm (with 650 nm reference filter) using a Microwell System Reader (Model 510, Organon Technica, Oss, The Netherlands).

### 2.5. Fluorescent Stains

After incubation for 48 h, live/dead staining was performed as previously described [25], by incubating the biofilms for 30 min at 37 °C with the green-fluorescent nucleic acid stain SYTO9 (Invitrogen Molecular Probes, Eugene, OR, USA) (3.4 µM; stock solution 5 mM in DMSO) combined with the red-fluorescent nucleic acid stain propidium iodide (PI, Sigma-Aldrich Chemie, GmbH, Steinheim, Germany) (27 µM; stock solution 20 mM in DMSO) in saline.

### 2.6. Confocal Microscopy

Microscopic examinations of biofilms in 8-well ibiTreat μ-Slides (Ibidi, Munich, Germany) were performed with a Zeiss LSM 710 Axio Observer Z1 laser scanning microscope equipped with a Plan Apochromat ×63/NA (numerical aperture) = 1.4 oil DIC (differential interference contrast) objective and a LD Plan-Neofluor ×40/NA = 0.6 Korr objective lens. To determine the structure of the biofilms, a series of horizontal (*xy*) optical sections were taken throughout the full length of the biofilm, and Z-stacks with 3 or 5 µm Z-steps were collected. Confocal images of green and red fluorescence were recorded simultaneously by CM using a multichannel mode. All of the instrument settings, such as excitation laser wavelength and (AOTF) Acousto-Optical Tunable Filters power percent setting, as well as detector gain, offset, and optical zoom, were identical among the experiments that were compared. Zeiss Zen 2.3 software (Carl Zeiss MicroImaging GmbH, Jena, Germany) was used for confocal image acquisition and processing, and image J software was used for analysis [25].

### 2.7. Statistical Analysis

Mean values and standard errors were calculated. Statistical significance was determined by Two-Factor Analysis of Variance (ANOVA) with Replication using Excel 2013.

## 3. Results

MIC (95–100% inhibition) for the S-8 compound and NA-8 was visually determined according to the CLSI recommendation, after 72 h (Table 1).

**Figure 1:**
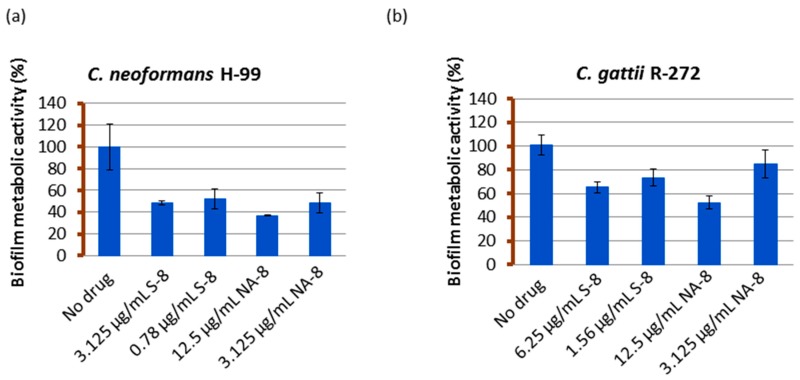
The effect of S-8 and NA-8 on biofilm metabolic activity. *C. neoformans* H-99 (**a**) and *C. gattii* R-272 (**b**) biofilms were grown in 96-well microtiter plates as described in the Methods section. The inhibition of biofilm formation by S-8 and NA-8 were examined by measuring metabolic activity (XTT assay). Mean ± standard error (SE).

Biofilms of both strains showed reductions in metabolic activity by XTT reduction assay when treated with sub-inhibitory concentrations of S-8 and NA-8 (1/4 and 1/16 of MIC) (Figure 1). The metabolic activity of *C. neoformans* H-99 biofilm was reduced by 51.6%, 47.8%, and 63.1%, 51.6% following incubation with S-8 and NA-8, respectively, and that of *C. gattii* R-272 was reduced to a lesser extent by 34.8%, 26.5% and 47.6%, 15.3%, respectively. Using the ANOVA F test, there was a significant difference between the effect of different substances on the production of biofilm for *C. neoformans* (F = 16.456, *p* < 0.001) and an almost significant difference for *C. gattii* (F = 4.980, *p* = 0.054).

**Figure 2:**
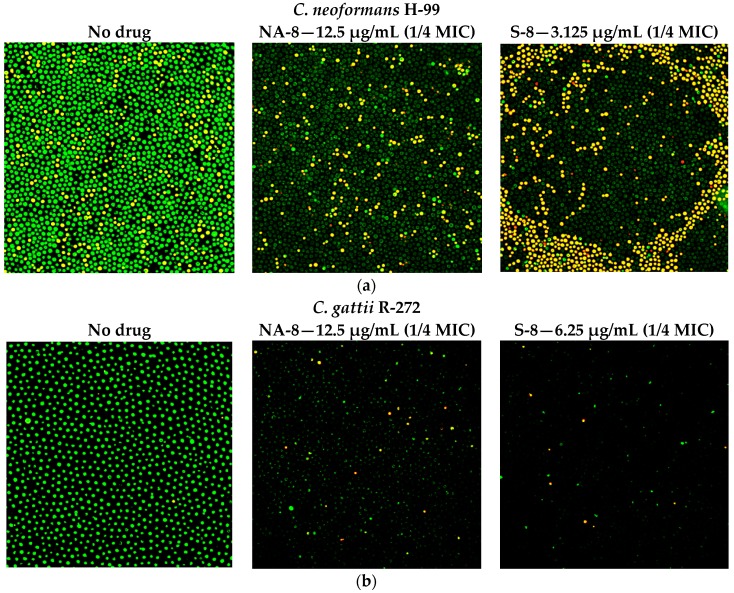
Effect of S-8 and NA-8 on the viability of *C. neoformans* and *C. gattii* biofilms. (**a**) Treatment of *C. neoformans* H-99 with S-8 and NA-8 during biofilm formation reduces nucleic acid staining by SYTO9. Treatment with S-8 also causes yeast death in the biofilm; (**b**) Treatment of *C. gattii* R-272 with S-8 and NA-8 during biofilm formation reduces nucleic acid staining by Syto9. Medium with 0.5% DMSO was used as a control. ×400. MIC, minimum inhibitory concentration. DMSO, dimethyl sulfoxide.

**Figure 3:**
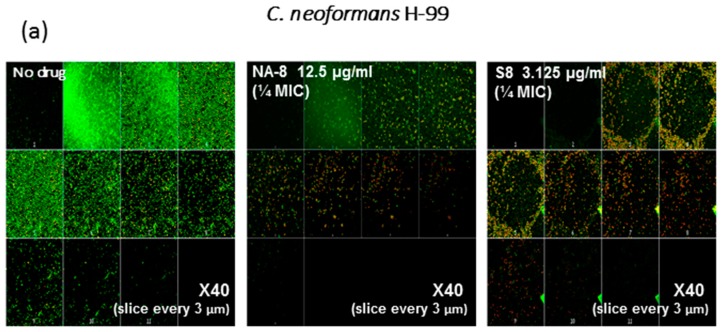

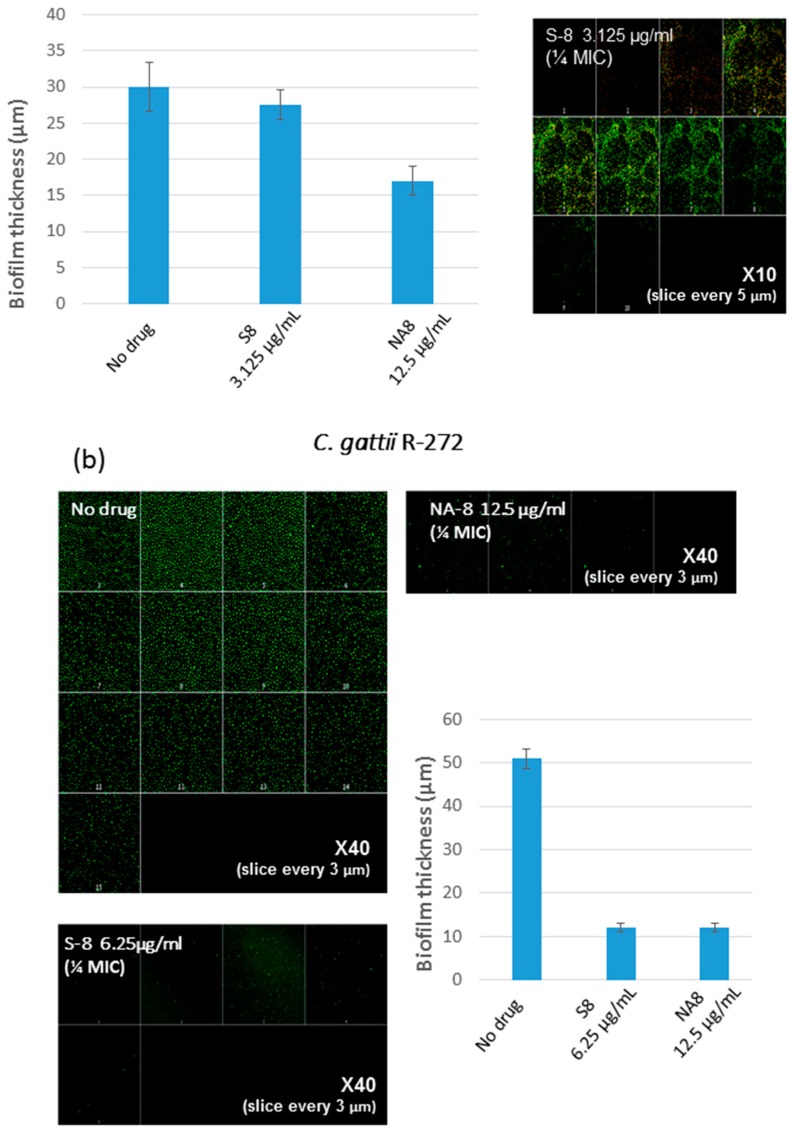
Effect of sub-inhibitory concentrations of S-8 and NA-8 on the biofilm thickness of *C. neoformans* (**a**) Treatment of *C. neoformans* H-99 during biofilm formation with NA-8 but not S-8 reduced biofilm thickness; (**b**) Treatment of *C. gattii* R-272 during biofilm formation with S-8 and NA-8 reduced biofilm thickness. Medium with 0.5% DMSO was used as a control. Mean ± SE. ×40.

CM examination was used to correlate the XTT reduction assay results with the visual effects on biofilm metabolism and structure (Figure 2). Regions of green fluorescence (Syto9) represent viable cells whereas yellow/red fluorescence (propidium iodide) represents nonviable cells. *C. neoformans* H-99 and *C. gattii* R-272 biofilms grown in the presence of media alone show regions of high metabolic activity, in contrast to the treatment of both strains with 1/4 MIC of S-8 and NA-8 during biofilm formation, that show reduced nucleic acid staining by Syto9, implicating yeast death in the biofilm. *C. neoformans* H-99 and *C. gattii* R-272 biofilms manifested a decrease in thickness and metabolic activity when treated with 1/4 MIC of S-8 and NA-8 as compared to the no drug control (Figure 3).

## 4. Discussion

The two novel heterocyclic compounds S-8 and NA-8 were found to be active against *Cryptococcus* biofilm at sub-MIC concentrations. Both compounds inhibited *C. neoformans* H-99 and *C. gattii* R-272 biofilm formation, as reflected by reduced metabolic activity and biofilm thickness. S-8 had a lethal effect on *C. neoformans*, although this effect was not observed with *C. gattii*. The biofilms were reduced by 50% following incubation with S-8 and NA-8 at a four-fold lower concentration than their MICs. Other antifungals, such as amphotericin B and fluconazole, affected biofilms only at concentrations 30- and 100-fold higher than their MICs for planktonic cells, respectively [14].

These antifungals are thought to exert their moderate anti-biofilm effect by interfering with the cryptococcal capsular main component, glucuronoxylomannan (GXM), which is necessary for adhesion to surfaces and for subsequent biofilm formation [5,10,30]. It is thought that the melanin deposited in cell walls of *C. neoformans* protects the yeast and reduces biofilm susceptibilities to these drugs [10,31], and that activation of QS and the coordination of the collective production of an exopolymeric matrix may act as a physical barrier that prevents the penetration of antifungal agents. Therefore, it is necessary to focus on QS inhibitors, such as TZD and SI, as anti-biofilm agents. Fungal biofilm integrity has been found to be dependent on QS, which is associated with biofilm formation and the increased pathogenicity of fungi [16]. QS in *C. neoformans* have been demonstrated to regulate biofilm cell growth, GXM release, and melanin synthesis [10]. The presence of pantothenic acid in *C. neoformans*-conditioned medium has been suggested to play a role in QS, causing an increase in biofilm growth and melanization [4].

Based on the cross-activity between QS molecules in bacteria and fungi [32,33,34,35], S-8 and NA-8 were found to be the most effective anti-biofilm agents against *C. albicans*, when tested among a series of novel thiazolidinedione (TZD) and succinimide (SI) derivatives (probable analogs/antagonists of *V. harveyi* autoinducer I) [25]. NA-8-treated cells were found to have multiple sterol rings that are similar to septin rings in *C. albicans* cells that are defective in sterol polarization (*C. albicans sur*7Δ mutant) and therefore have disrupted cell wall synthesis, polystyrene adhesion, and biofilm formation [26,27,28]. Sterol rings induced by NA-8 may have indicated a disruption to the polarization of lipid rafts that anchor and localize cell wall proteins in *C. albicans*, leading to defects in cell wall morphology, and cell–cell/cell–substrate adhesion, thus inhibiting *Candida* biofilm formation [25]. S-8, a novel bacterial QS quencher thiazolidinedione derivative, exhibited specific anti-biofilm and anti-adhesion activity against *C. albicans* at concentrations four- to eight-fold lower than the MIC [36]. It disrupted fungal morphogenesis by inhibiting the transition of yeasts to hyphal forms and the modulation of cell wall hydrophobicity. Genes associated with biofilm formation, adhesion, and filamentation were found to be downregulated by S-8 in a dose-dependent fashion, suggesting its therapeutic potential in the treatment and prevention of biofilm-associated infections.

Whether the biofilm inhibitory properties of S-8 and NA-8 in this work may be related to disruption in cell wall morphology and substrate adhesion in a similar way as to its effect on *C. albicans* biofilm remains to be determined. A recently published genome-scale chemical genetic data map that quantified the impact of small molecules on 1448 *C. neoformans* gene knockouts points to S-8 as a trigger of G2/M arrest, possibly through the inhibition of Cdc25 phosphatase. This supports the model that S-8 inhibits *C. neoformans* growth through the cell cycle at least in part via the inhibition of Cdc25 phosphatase. Whether this explains the impact of S-8 on biofilms requires further investigation, and an ultimate proof that Cdc25 is the target of S-8 requires the isolation of resistance alleles of *Cdc-25* [29].

We believe that S-8 and NA-8 compounds are potential anti-biofilm agents that may have wide use. However, further investigation of the effects of these compounds on gene and protein expression as well as of the ways in which specific pathways are affected is warranted.

## Figures and Tables

**Table 1:** Susceptibility of *C. neoformans* and *C. gattii* to S-8 and NA-8.

Compound and Structure	MIC ^a^ (µg/mL)	
	*C. neoformans* H-99	*C. gattii* R-272
S-8 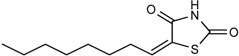	12.5	25
NA-8 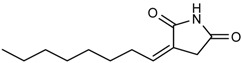	50	50

^a^ Minimum inhibitory concentration.
